# Influence of motivation and academic performance in the use of Augmented Reality in education. A systematic review

**DOI:** 10.3389/fpsyg.2022.1011409

**Published:** 2022-10-11

**Authors:** Antonio Amores-Valencia, Daniel Burgos, John W. Branch-Bedoya

**Affiliations:** ^1^Research Institute for Innovation and Technology in Education (UNIR iTED), Universidad Internacional de La Rioja, Logroño, Spain; ^2^Facultad de Minas, Universidad Nacional de Colombia, Medellín, Colombia

**Keywords:** Augmented Reality, motivation, academic performance, high school, systematic review

## Abstract

The recent technologies rise today as a tool of significant importance today, especially in the educational context. In this sense, Augmented Reality (AR) is a technology that is achieving a greater presence in educational centers in the last decade. However, Augmented Reality has not been explored in depth at the Secondary Education stage. Due to this, it is essential to analyze and concentrate the scientific research developed around this educational technology at that stage. Therefore, the aim of this research is to describe the influence that Augmented Reality shows on the motivation and academic performance of students in the Secondary Education stage. In relation to the methodology, a systematic review of the literature has been conducted using the Kitchenham protocol, where several factors have been analyzed, such as subjects, activities, and electronic implementation devices, together with the effects on motivation and student's academic performance. The Scopus and Web of Science (WoS) databases have been used to search for scientific papers, with a total of 344 investigations being analyzed between 2012 and 2022. The methodological stages considered were the formulation of research questions, the choice of data sources, search strategies, inclusion and exclusion criteria and quality assessment, and finally, data extraction and synthesis. The results obtained have shown that the use of AR in the classroom provides higher levels of motivation, reflected by factors such as attention, relevance, confidence, and satisfaction, and reflects better results in the tests carried out on the experimental groups compared to the control groups, which means an improvement in the academic performance of students. These results supply a fundamental theoretical basis, where the different teachers should be supported for the incorporation of AR in the classroom, since how this educational technology has been shown offers great opportunities. Likewise, the development of research in areas not so addressed can further clarify the generality of AR based on its influence on learning. In addition, the fields of natural sciences and logical-mathematical have been the most addressed, managing to implement their contents through object modeling. In short, this research highlights the importance of incorporating Augmented Reality into all areas and educational stages, since it is a significant improvement in the teaching and learning process.

## Introduction

Today, the use of technological devices is present in most of the activities that people conduct daily. This is largely due to the introduction of technology in the development and implementation process in many fields (Huang and Liao, 2017; Juan, 2019; McLean and Wilson, 2019; Schaffernak et al., 2020; Rezaee et al., 2021). In this sense, education cannot be relegated to the background, much less leave aside the new devices and existing technological tools (Macías-González and Manresa-Yee, 2013). For this reason, the teaching and learning process must adapt to today's society, to work in parallel with the demands of an increasingly changing market.

Under this premise, Information and Communication Technologies (ICT) are the tools that have brought about a great transformation of daily life, whatever the aspect that may be glimpsed. This fact, together with the creation of new jobs, requires the obligation of continuous training, which produces people fully prepared for the future changes that lie ahead (Cabero-Almenara, 2015). In addition, the introduction of educational technology supposes an enhancement of student motivation, which translates into better academic performance (Area-Moreira et al., 2018).

On the other hand, the little or no use of technological tools in the classroom shows very worrying levels of motivation, especially in Secondary Education, so teachers must reconsider their position in search of a more appropriate environment for the reality of their needs students (Amores-Valencia and De-Casas-Moreno, 2019). According to Hernandez (2017) the lack of motivation of the students is not only an obstacle for the learning of concepts, but also a problem in the daily teaching work of the teachers, because the students are inattentive, and it often generates disorder. In this negative environment, educational technologies play a significant role because they can be seen as a powerful motivational tool (Vedadi et al., 2017). In addition, it is essential to train students in critical thinking, emphasizing the reflective part, as well as giving greater efforts in the face of motivational and emotional difficulties such as low expectations, disinterest and high pressure from students (Barroso-Osuna and Cabero-Almenara, 2013).

One of the most attractive, dynamic and interactive proposals is the integration of Augmented Reality in the classroom, as shown by numerous studies in recent years. In reference to the origin of the concept, it was Azuma (1997) who defined it as a technology that enhances the sensory uptake of people since it can combine real and virtual elements in an interactive scenario in real time. In this sense, the implementation of Augmented Reality requires working with technological devices such as tablets, smartphones, and computers that generate interaction between users, producing empathic experiences (Cabero-Almenara and Barroso-Osuna, 2016b). In addition, with the introduction of new active methodologies that will affect students in quite diverse ways in face-to-face, online and blended learning and teaching contexts (Buchner, 2021).

This work is organized as follows. The first section shows the research related to this work, specifically those that bring together Augmented Reality with motivation and academic performance in Secondary Education. The second section presents the method developed where each of the phases is broken down. The third section shows the results obtained through an exhaustive analysis of the information. In the fourth section, the research questions are discussed and answered. Finally, the relevant conclusions are developed based on the contrast of the aims set, and the results obtained.

### Theoretical framework

In this sense, Augmented Reality (AR) is the technology with the greatest effect on education in recent years, enabling the coordination of the real and the virtual at the same time (Cabero-Almenara and Barroso-Osuna, 2016a). In addition, it helps to create more motivating and attractive teaching and learning scenarios that, in turn, would be impossible to conduct in the real world (Duh and Klopher, 2013; Wojciechowski and Cellary, 2013; Huang et al., 2016). Likewise, the use of this educational technology can contribute positively to the interest, motivation and performance of students (Di Serio et al., 2013 Redondo-Domínguez et al., 2014; Sommerauer and Müller, 2014; Reinoso-Peinado, 2016).

In this way, many investigations highlight the effect and influence that Augmented Reality has and will have on teaching and learning processes (Yuen et al., 2013; Bower et al., 2014; Ibáñez et al., 2014; Jerábek et al., 2014; Coimbra et al., 2015; Solak and Cakir, 2015; Fonseca-Escudero et al., 2016; Marín-Díaz, 2016; Sánchez-Bolado, 2017; Cabero-Almenara and Marín-Díaz, 2018). In addition, some scientific works are related to games, applications and illustrated books, which use AR to show new ways of learning, incorporating animations of images and videos to the illustration of the texts (Lin et al., 2017; Álvarez-Marín et al., 2020).

On the other hand, most research has considered university students, where extra motivation is predisposed as it is a non-compulsory educational stage (Fernández-Robles, 2017; Barroso-Osuna et al., 2018; Marín-Díaz et al., 2018; Gómez-García et al., 2020). Another profile of the student body studied has been primary school students where performance and motivation tend to have high values (Toledo-Morales and Sánchez-García, 2017; Wang, 2017; Kirikkaya and Başgül, 2019; Lai et al., 2019; López-Belmonte et al., 2019).

Other fundamental aspects related to motivation are consciousness and emotions. According to Collazos et al. (2021) teachers must know when and how to participate, in such a way that they adapt to the situation of each group of students, and in the opposite case, the students must be aware of what is happening in their environment with the purpose of achieve proper interaction between all members. Along the same lines, Mestre-Navas et al. (2017) shows that emotional intelligence is closely related to motivation, since it supplies the necessary skills to control one's own and others' emotions, which produces better learning. For this, it is essential that emotions are perceived, found, valued and expressed adequately and precisely. In this sense, assimilating emotions is a vital step to achieve a good understanding of knowledge. Likewise, the organization of emotions brings with it a cognitive effort, which, guided by the teacher, produces an adequate management of emotions in real-life situations.

Finally, the integration of Augmented Reality in the educational field is a fact confirmed in the multiple investigations previously developed, however, it is essential to know the influence of the gender of the students in the results obtained when this educational technology is used (Abad-Segura et al., 2020; Álvarez-Marín et al., 2020). In this sense, there is a multitude of investigations that try to find out the repercussion that gender shows according to motivation and interest, the acceptance of the use of Augmented Reality as an educational tool and the acquisition of knowledge or performance (Hsu, 2019; López-García et al., 2019). For this reason, this first section will specify the results and conclusions that have been described by the different investigations on the influence that the gender of the students shows in motivation, in the acceptance and use of learning objects and in the acquisition of knowledge, and before the use of Augmented Reality in the different educational stages.

To know what other authors have investigated about how Augmented Reality influences the motivation and performance of students in the various learning and teaching contexts mentioned, a systematic review of the literature has been carried out, based on studies that include different techniques to show this technology. Specifically, it has focused on Secondary Education, since it shows the worst rates of motivation and academic performance of students (Amores-Valencia and De-Casas-Moreno, 2019). The studies included in this systematic review have been obtained from the Scopus and Web of Science (WoS) databases, where a series of keywords have been introduced, to later discard repeated documents and keep those that met the criteria of inclusion. Inclusion and exclusion detailed in the method.

### Augmented Reality in education

Regarding the contributions made by Augmented Reality to the educational field, the considerable number of applications developed to work with this educational technology in the classroom in the different subjects stand out. However, the use of this educational technology is still very restricted due to the lack of teacher training, since teachers who wish to integrate Augmented Reality in their classrooms must get the knowledge in a self-taught way, during non-school hours and on many occasions without adequate resources (González-Segredo and Hernández-Cabrera, 2022).

On the other hand, said technology presents a series of fundamental characteristics for the new educational paradigm, since it provides interaction with resources, visualization of information or creation of scenarios that enhance the understanding of concepts, which has repercussions on the learning process (Aguilar-Acevedo et al., 2022). Another characteristic that Augmented Reality grants is the choice of applying active methodologies, where the teacher plays the role of guide and the students take an active role, managing their own learning. In addition, the student imposes his own rhythm, which eases the process of understanding and getting knowledge. All of this provides autonomous learning, where the student usually shows more interest than with traditional methodologies (Amores-Valencia, 2020).

According to Cabero-Almenara and Barroso-Osuna (2016b), the relevance of Augmented Reality in the future will be especially important since it combines reality with virtuality at the same time and place. In this sense, Castro-Marcos (2022) points out that this educational technology gives the possibility of creating learning scenarios that would be impossible to develop in the real world. According to this author, these more attractive scenarios enhance the interest and motivation of the student, an aspect that undoubtedly affects their academic performance.

In this way, many investigations highlight the impact that Augmented Reality will have on teaching and learning processes (Fonseca-Escudero et al., 2016; Marín-Díaz, 2016; Sánchez-Bolado, 2017). In addition, some scientific works are related to games, applications and illustrated books, which use Augmented Reality to show new forms of learning, incorporating animations of images and videos to the illustration of the texts (De-Paiva-Guimarães and Farinazzo-Martins, 2014; Lin et al., 2017; Álvarez-Marín et al., 2020).

Other applications of Augmented Reality are focused on the professional field since virtual scenarios can be created and implemented that do not pose a danger or physical harm to people. Thus, a safe and at the same time adequate training can be conducted, where the student's skills are experienced in virtual situations (Akçayir et al., 2016). This feature gives companies a fantastic opportunity to train their employees, guaranteeing their health (Carlton, 2017). Likewise, with the rise of e-learning training, Augmented Reality has made it possible to work on practical content, enabling the acquisition of knowledge that would only have been possible in face-to-face training (Reinoso-Peinado, 2016).

In reference to the acceptance and use of Augmented Reality learning objects, the authors Wang et al. (2017) and Bursztyn et al. (2020) state that no significant difference is observed between male and female students. These investigations show that factors such as perceived enjoyment, perceived usefulness and perceived ease of use show practically identical values in men and women. Although it is true that one cannot speak of the absolute non-existence of gender disparity, since some research has highlighted that said difference exists, emphasizing the real and palpable difference in the perception that students present in the use of Augmented Reality based on gender (Dirin et al., 2019; Park et al., 2019). This fact indicates that the digital divide between genders continues to exist, although fortunately over the years it is being reduced (Cabero-Almenara et al., 2019b). Thanks to the effort of educational centers to equate all students in an identical way, their digital competence is not related to gender (Hohlfeld et al., 2013).

In conclusion, Cabero-Almenara and Marín-Díaz (2018) show a series of educational possibilities of Augmented Reality:

- It improves the real contents easing its understanding.
- Develop multimedia training environments.
- Promotes online learning.
- Cut non-relevant information that hinders the acquisition of knowledge.
- Create safe learning scenarios.
- Helps active and productive learning of Augmented Reality resources.
- Incorporate extra information in the form of illustrations, videos, or audios.
- Design more attractive simulators for learning.
- It eases the visualization of contents from various perspectives.
- It promotes the use of active strategies or methodologies.

### Motivation and academic performance

For many years, cognitive variables have been the most analyzed in the learning process, but in the 1990's there was an increase in the number of studies seeking the influence of the motivational aspect in the design of congruent models that explain academic performance (Pressley et al., 1992; Pintrich et al., 1993; Borkowski, 1994; García and Pintrich, 1994; Pintrich, 1994; Schunk and Zimmennan, 1994; García, 1995; Boekaerts, 1996). However, for these authors, there must be a relationship between the cognitive and the motivational to obtain an improvement in academic performance.

Motivation tries to activate student behavior aimed at achievements and goals if there is an effort behind it during the process. For this reason, motivation encompasses many variables such as expectations of achievement, relative attributions, self-value, self-esteem and self-concept (González-Pienda, 2003). In this sense, Weiner (1986) states that motivated behavior is obtained based on the possibilities of achieving goals and their value. These two components manage the success or failure of a student and are set up by the relative attributions that the student has of himself. Therefore, Weiner (1985, 1986) defines attributions as the greatest determinant of motivation in terms of the results and academic performance of the student.

In reference to self-concept, the research conducted focused on the student's academic behavior, due to the importance of knowing how academic goals are obtained and in what context they are achieved (González-Pienda et al., 1997). These studies confirmed the relationship between self-concept and the academic performance of students, however, there are still doubts between the processes that make this relationship possible and its directionality. In such a way that there is research that corroborates the reciprocity between self-concept and academic performance (Marsh and Yeung, 1997) and others that expose the unidirectionality of performance on the self-concept of students (Helmke and van Aken, 1995).

According to Núñez (2009), the skills and competencies of a student are not enough to improve their academic performance, but it is necessary to consider their motivation. This statement comes to extol the importance of motivation in a student's performance, since it does not depend exclusively on the knowledge and skills that he has. Similarly, Garrido-Macías et al. (2013) states that the effect of motivation on a student's academic performance grows the higher their self-esteem and their assessment of tasks.

For all that has happened, the motivational variables that are subject to the types of motivation are directly related to academic performance and are decisive in the educational process (Mascarenhas et al., 2005; Alonso-Tapia and Ruiz-Díaz, 2007; Martín et al., 2008; Miñano-Pérez and Castejón-Costa, 2011; Barca-Lozano et al., 2012). On this occasion, it can be affirmed that intrinsic and extrinsic motivation can be addressed jointly or separately, since they are not opposed based on the academic performance presented by students (Usán-Supervía and Salavera-Bordás, 2018). Therefore, it is vital to continue analyzing the relationship between motivation and academic performance, to learn more about the interrelated factors and thus improve the teaching and learning process (Jerez-Carrillo, 2021).

Finally, the motivation that students present in relation to their gender is reflected in the different investigations carried out by Hanafi et al. (2017) and Buchner (2021), where it is stated that the behavior that students present is different from that the students present and, therefore, a significant difference in motivation is appreciated before the use of Augmented Reality. However, other authors indicate that the difference shown is negligible to be considered (Bursztyn et al., 2017; López-Belmonte et al., 2019). Regarding academic performance, Del-Rio-Guerra et al. (2019) and Gómez-Tone et al. (2020) state that students and students show the same results when Augmented Reality is applied in classrooms. This means that the academic performance of students is invariant based on their gender. This information is valuable since it gives teachers the possibility of implementing this educational technology without the need to take this feature into account. However, one must always be aware of the disparity that exists in each of the classrooms, since sometimes a differentiation can be found between male and female students in the acquisition of knowledge (Chen et al., 2021).

The bibliographical reviews on the use of AR in Secondary Education reported up to now have almost never brought together two of the great educational factors, such as motivation and academic performance (Martín-Gutiérrez and Meneses-Fernández, 2014; Liu et al., 2019). For this reason, this research aims to analyze the influence that Augmented Reality shows on the motivation and academic performance of students in the Secondary Education stage.

## Methods

### General guidelines

This research has been developed through the systematic review of the literature (RSL) process, based on the proposal of Kitchenham and Charters (2007). According to the authors, this protocol requires an exhaustive, objective, and reliable general description, which is governed by defined and strict steps. Specifically, the steps followed for the development of the systematic review were the following:

1. Planning the review
  - Identification of the need for a review
  - Specifying the research questions

2. Conducting the review
  - Identification of data sources
  - Selection of search strategies
  - Inclusion and exclusion criteria
  - Study quality assessment
  - Data extraction
  - Data synthesis

3. Results report
  - Included and Excluded Studies
  - Interpretation of results
  - Formatting the report

In this way, the analysis of the literature has been developed under the recommendations of the Preferred Reporting Items for Systematic Reviews and Meta-Analysis (PRISMA; Urrútia and Bonfill, 2010; Moher et al., 2015).

### Planning the review

Given the research studies analyzed, where the factors of motivation and academic performance have been worked on individually, it is necessary to develop a work where these factors are grouped and detailed, particularizing in the Secondary Education stage, since it is about a stage where the lowest levels are shown (Picó-Lozano, 2014; González-Valenzuela and Martín Ruiz, 2019; Jerez-Carrillo, 2021). For this reason, the investigations that combine these two dimensions have been brought together, in such a way that the following research question can be answered:

What is the status of the use of Augmented Reality through markers in terms of population, interventions, comparators, results and study designs, considering studies between 2012 and 2022 included in two interdisciplinary databases: Scopus and Web of Science, in order to know the impact on motivation and academic performance in students of the Secondary Education stage?

According to this main research question, a series of research sub-questions were defined:

RQ1: What subjects and groups are the recipients of educational activities with AR?

RQ2: What technological devices have been used to generate and/or run AR applications?

RQ3: How are educational activities implemented with AR in the classroom?

RQ4: What motivational impact do students have based on the use of AR?

RQ5: How does the use of AR influence the academic performance of students?

### Conducting the review

#### Identification of data sources

The search for jobs was conducted using the Scopus and Web of Science (WoS) databases, relevant scientific content platforms, since they bring together a multitude of scientific publications from various areas of knowledge. Specifically, they host a multitude of works related to Augmented Reality in secondary education.

On the other hand, these two databases allow searching in advanced structures thanks to the use of logical operators, which fit perfectly to the particularities of the systematic review proposed in this research. In addition, the use of filtering tools and bibliometric analysis provide excellent information to the work presented.

#### Selection of search strategies

The search strategies are one of the high points of the research, since the information available in the databases must be filtered, in such a way that the selected works allow answering the research questions posed and, consequently, fulfilling the marked target. According to Kitchenham et al. (2009) search strategies make it possible to assess the integrity of the information search.

In reference to this premise, the search strings were defined in such a way that the defined keywords could be reached, and in turn answer the research questions raised.

The structured search used to search for jobs was conducted on July 14, 2022, and followed the following format according to each database:

The search string adapted to the syntax required by the Scientific Information Institute-Scopus database was as follows: (TITLE-ABS-KEY (“Augmented Reality” OR “augmenting reality” OR “AR”) AND TITLE-ABS -KEY ((“motivation” OR “performance)) AND TITLE-ABS-KEY (“education”)) AND DOCTYPE (ar OR cp) AND PUBYEAR > 2011 AND PUBYEAR < 2022 AND (LIMIT-TO (LANGUAGE, “English”)).

On the other hand, the search string adapted to the syntax required by the Institute for Scientific Information-Web of Science database was as follows: TOPIC: ((“Augmented Reality” OR “Augmenting reality” OR “AR”)) AND TOPIC ((motivation OR “academic performance”)) AND TOPIC: ((“education”)) AND YEAR PUBLISHED (2012–2021). Refined by: LANGUAGES: (ENGLISH) AND TYPES OF DOCUMENTS: (ARTICLE).

#### Inclusion and exclusion criteria

In the selection of studies, those works that meet the conditions to be considered in the RSL are chosen, under the premise of the inclusion and exclusion criteria. In the study selection process, works were included in which the areas of application, target groups, technological tools used, motivation and academic performance could be identified. In addition, the duplication of references and their subsequent elimination was conducted using Microsoft Excel software.

According to Kitchenham and Charters (2007), studies can be selected by title and abstract, obtaining a complete copy of them. Based on these suggestions, the study selection criteria were detailed, which in turn included the keywords and search strings defined from the research questions:

- In the title and abstract, the sequence of words “Augmented Reality” or “augmenting reality” or “AR” should be included.
- The abstract must contain the sequence of words “high school.”
- And in the summary the term “motivation” or “academic performance” should appear, or both words at the same time.

The eligibility criterion taken to include and exclude studies was if the word appeared, it was marked with the number 1, otherwise, it was indicated with the number 0. However, in cases where the title and abstract were not enough to determine its inclusion or exclusion, the authors evaluated all the content of the work.

To clarify the selection criteria, the following function was detailed in the Microsoft Excel software:

IF(AND(TITLE=1;ABSTRACT=1; COUNT.IF(ABSTRACT:ABSTRACT;1)≥1);”candidate article;” “no”).

#### Study quality assessment

One of the most relevant sections of the systematic review is the evaluation of the quality of the study, since it involves determining those works that allow an adequate response to the research questions and, therefore, fulfill the stated objective. For this reason, it is necessary to analyse the results without any type of interference and mistake, counting on the appropriate studies for the proposal (Carrizo and Moller, 2018).

According to the authors Kitchenham and Charters (2007), quality verification questions must be defined. Therefore, a questionnaire was designed based on seven items that outline the quality of the study, in such a way that they were scored to know a general measure of the quality of the selected works. In this sense, the questions were adapted to the present study, and determined the relevance of these works around the deepening toward a complete reading and subsequent analysis.

The quality questions that are developed below allowed minimizing the bias of the study and maximizing both external and internal validity.

- Are application areas and target groups established in Secondary Education?
- Does the document describe the electronic devices and technological applications used for educational activities with AR?
- Does the document indicate the form of RA application conducted?
- Were the users who participated in the creation of content fully defined?
- Is the contribution of AR to student motivation clearly described and defined?
- Is achievement included as one of the main contributions of AR in Secondary Education?
- Are all research questions answered?

The quality assessment checklist describes the score based on the quality level of the article. Each of the questions was evaluated using the following information (Table 1).

**Table 1:** Quality assessment checklist.

**Level**	**Description**	**Score**
Si	Information is explicitly defined/evaluated	1
Partially	Information is implicit/stated	0.5
No	Information is not inferable	0

Articles were included and classified as “full reading article” in the following stages if the sum of the criteria was >4 points.

#### Data extraction

The software used to manage the data and analyse the information of the selected works were Mendeley and Microsoft Excel.

In the case of Microsoft Excel, it was used to manage the articles resulting from the search in the scientific databases, eliminate duplicate references and classify the information of each article. The workbook is made up of several sheets, where each of the phases is documented.

With respect to Mendeley, it was used to bring together the candidate articles, and highlight the highlighted information, underlining with a different color depending on the category.

The data extraction process was developed in three stages.

- Information analysis: the analysis and classification of the article information was conducted from the bottom up. The text fragments that answer the research questions were highlighted with different colors, using the Mendeley tool. This action allowed further reading and detailed analysis and classification.
- Information classification: label codes to assign a representative meaning to the highlighted elements. The information was defined synchronously with the Information Analysis stage. Table 2 shows the codes considered for each of the research questions.
- Information extraction: Each text segment highlighted in the information analysis stage is classified according to the code established in the classification stage. A spreadsheet is needed to process the information generated at this stage https://alumnosunir-my.sharepoint.com/:x:/g/personal/antoniojesus_amores916_comunidadunir_net/EY58lmoHHVpMvxPUV5FVKj8BB-NvKu_65vT66VQ6G5Cuvg?rtime=udITTMSg2kg.

**Table 2:** Acronyms to classify information.

**Source**	**Acronym**
Application areas or subjects	Biology (B)—Mathematic (M)—Languages (L)—Technology (T)—Physical Education (PE)—History (H)—Chemistry (C)—Health Education (HE)
Target groups	7° Grade (7G)–12–13 years 8° Grade (8G)–13–14 years 9° Grade (9G)–14–15 years 10° Grade (10G)–15–16 years 11° Grade (11G)–16–17 years 12° Grade (12G)–17–18 years
AR activities in educational settings	Discovery-based Learning (DL)—Objects Modeling (OM)—AR Books (B)—Skills Training (ST)—AR Gaming (G)
Electronic device	Computer (C)—Mobile Phone (MP)—Tablet (T)—Glasse (G)
AR technology software or application	Lightining Studios (LS)—Unity/Vuforia (UV)—ARDehaes toolkit (AT)—Aurasma (A)—RAVVAR (R)—Metaverse Studio (MS)
Content creation	Designed by students (DS)—Designed by teacher (DT)—Designed by external persons (DE)
Motivational level	Very high (VH)—High (H)—Medium (M)—Low (L)—Very Low (VL)
Academic performance level	Very high (VH)—High (H)—Medium (M)—Low (L)—Very Low (VL)

#### Data synthesis

The data was tabulated and displayed to represent:

- The different areas or subjects of application and target groups that participated in the articles.
- Directions of Augmented Reality in educational activities.
- Electronic devices, applications and software used in the different investigations.
- The creation of educational activities using Augmented Reality.
- Level of motivation observed in the articles.
- Degree of performance obtained thanks to the use of Augmented Reality.

## Results

This section is structured in response to the research questions, after going through the analysis process developed in the previous section. For this, the protocol chosen during data extraction has been considered. Further, data extracted from the review protocol are consolidated in the spreadsheet: https://bit.ly/3znd49h.

### Included and excluded studies

This first section breaks down the results obtained thanks to the search strings entered and the inclusion and exclusion criteria developed.

The first step was to introduce specific search strings based on each of the scientific databases used. This process resulted in a multitude of investigations, Table 3 shows the records obtained:

**Table 3:** Records obtained.

**Criteria**	**Filters**	**Scopus**	**Web of Science (WoS)**
Restriction	Topic (title, abstract, and keywords)	621	634
Period	2012–2022	536	547
Document type	Articles and conference proceedings	473	348
Language	English	440	285
Eligibility	High School	171	173
**Total**		344

It should be noted that 344 results were obtained between both databases, and the search in them occurred on July 14, 2022.

The second step was to eliminate the duplication of investigations that could be seen in both databases. For this, the Microsoft Excel tool was used, where the number of scientific articles was reduced to a total of 258 works. Next, the eligibility criteria based on incorrect titles and abstracts were considered, excluding a total of 215 investigations based on this criterion.

The investigations selected once the eligibility criteria of the systematic review were addressed are described in Table 4.

**Table 4:** Number of investigations chosen.

**Criteria**	**Papers**
Elected papers	43
Excluded papers	215

The investigations selected once the quality assessment of the systematic review was addressed are described in Table 5.

**Table 5:** Full reading articles included.

**Criteria**	**Papers**
Full reading papers	13
Excluded papers	30

**Figure 1:**
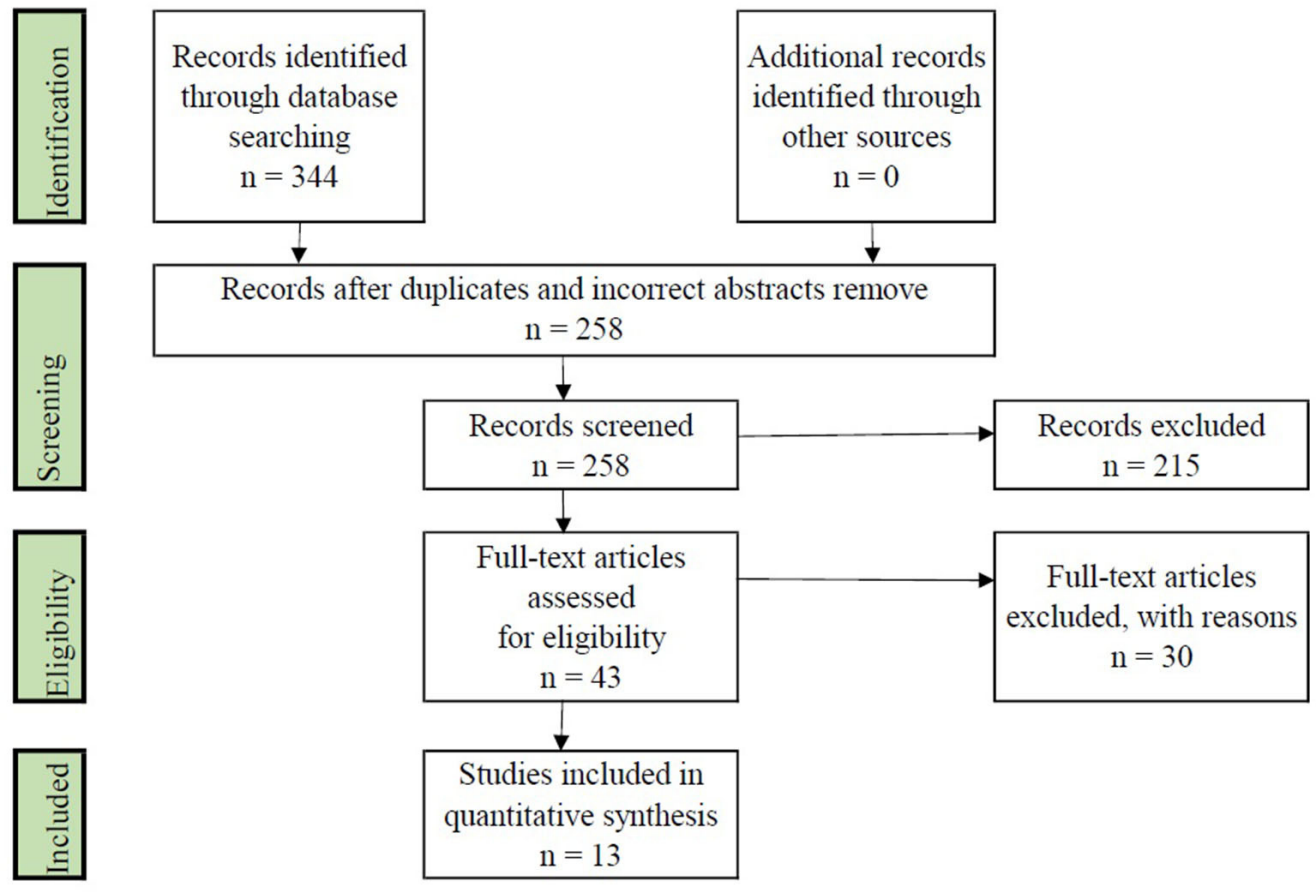
Selection process of the studies.

Figure 1 presents the phases and results of the number of scientific works that have been carried out in the process of systematic review of the literature, following a process of identification, review, eligibility, and inclusion (Moher et al., 2009).

### Interpretation of results

In the first place, the number of published articles, based on the characteristics that are worked on in this systematic review, has obtained rapid growth in the years 2019, 2020, and 2021. Another of the fields that has been analyzed has been the origin of the scientific articles, where six (6) countries of origin have been found, of which Taiwan stands out with seven (7) publications. Finally, the works analyzed are concentrated in nine (9) journals and four (4) conference proceedings, which is 69 and 31%, respectively.

Next, the results obtained around the designed research questions are detailed, considering the evaluation of the articles together with the information analysis process.

#### Target areas and groups

**Figure 2:**
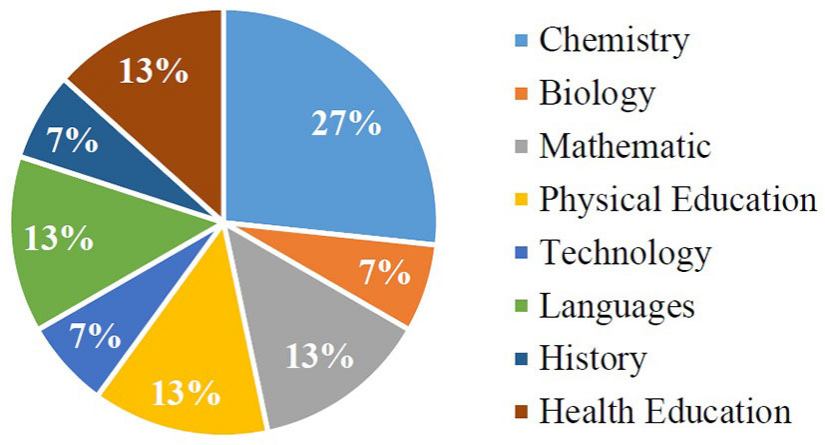
Application areas or subjects in Augmented Reality.

Since the systematic review is developed in the educational stage of Secondary Education, the areas that appear are relevant in that phase. In this way, Figure 2 shows the different subjects where Augmented Reality has been implemented in the classroom.

Regarding the target groups of the activities with Augmented Reality, they are concentrated in the courses included in Secondary Education, that is, from 7 to 12th grade. In this sense, the students included in the 10 and 11th courses, whose ages range between 15 and 17 years, have accounted for 32.5% each. On the contrary, the 8 and 9th grades, whose ages vary between 13 and 15 years, have occupied 17.5% each. In short, students in the last years of Secondary Education have been exposed to investigations with Augmented Reality in 65% of the total cases investigated.

#### Technological devices and applications

**Figure 3:**
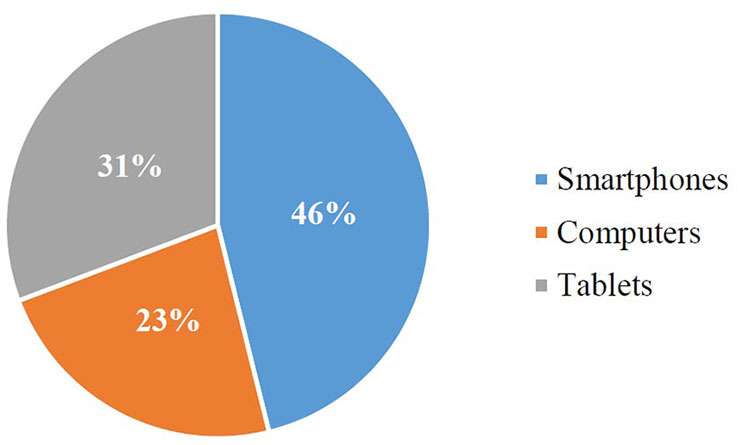
Electronic devices used.

The use of electronic devices for the implementation of educational activities based on Augmented Reality is a mandatory measure for this type of research. Based on this, the results obtained are shown in Figure 3.

As for the different technologies that have been used to create applications or software for Augmented Reality, Vuforia stands out. This development kit has been used by 54% of all developers, being its multiplatform engine Unity. The rest of the investigations have used other tools equally, which has meant 7.6% each. These applications are the following: Lighting Studios, ARDehaes toolkit, Aurasma, RAVVAR, and Metaverse Studio.

#### Design and forms of AR application

In reference to the design of educational activities based on Augmented Reality, it has been entirely developed by people outside the teaching staff or students who have been part of the research. In all cases, 100% of the researchers have overseen creating the content that was later applied in the different classrooms. Therefore, the role that teachers have developed has been that of guide or facilitator of the information necessary to conduct research practices. In this sense, they have had to deepen or conduct training tasks around the different Augmented Reality applications that have been implemented, in order to take them to the classroom.

On the other hand, the implementation of these contents has been conducted through different forms of application of Augmented Reality. In this sense, object modeling has been present in most of the investigations, accounting for 45.45% of the total. This was followed by AR books with 18.18% of the total, although closely followed by gamification of educational games and skills training with 13.63% of the total each. Finally, discovery-based learning techniques have been applied, accounting for 9.11% of the total.

#### Motivation and academic performance

Before presenting the results obtained in each of the categories, it is necessary to mention that these variables have been analyzed globally, leaving aside the subject of application, the target group, the methodology implemented, and the technological devices and applications used.

The first category represents the motivation shown by students during the process of implementation and evaluation of educational activities based on Augmented Reality. On this occasion, the different works have been analyzed to find out what levels of motivation or interest have been shown by the students who have used this educational technology compared to the students who have not used it. To do this, the Instructional Materials for Motivation (IMMS) instrument has been used to determine the indicators of attention, relevance, trust, and satisfaction on which Keller's ARCS model (1987, 2010) is based. The data obtained from this systematic review of the literature reflect that motivation levels have grown ostensibly. Specifically, 83.33% of the investigations grant an increase in the degree of interest and motivation in the students. Likewise, the works investigated have indicated through interviews or questionnaires that Augmented Reality has increased the predisposition and interest in the teaching and learning process.

The second category indicates the impact that the use of Augmented Reality has had on students in the acquisition of knowledge and therefore in their qualifications. On this occasion, it was about evaluating the knowledge acquired by the students, once this technology has been implemented in the classrooms, by means of tests or tests. In this way, the qualifications of the students have been contrasted with the purpose of obtaining an assessment of the importance of the use of this educational technology. In this sense, 77% of the works analyzed have refuted that the use of Augmented Reality has improved the grades of the students. In addition, no research work has stated that the use of this technology in the teaching and learning process has led to a drop in grades.

## Discussion

In this section, the results analyzed are discussed, the research questions are answered based on the findings, and finally the conclusions drawn from this systematic review of the literature are presented.

RQ1: What subjects and groups are the recipients of educational activities with AR?

At first, it is sought that Augmented Reality has been applied only to the Secondary Education stage. This fact requires that the subjects that appear in the investigations be worked on in the curriculum of said educational stage. However, the spectrum of areas in this stage is very broad, however, the data obtained reflect that the predominant subjects are grouped in natural sciences and logical-mathematical (Chen and Liao, 2015; Lin et al., 2015; Wang et al., 2017; Chen and Chen, 2018; Hsieh and Chen, 2019; Cen et al., 2020; Tarng et al., 2021). Proof of this are the subjects of Chemistry, Biology and Mathematics, which make up more than 50% of the studies analyzed. In these investigations, the contribution of Augmented Reality is highlighted based on the visualization, understanding and acquisition of content about these subjects, which brings with it an increase in student attention during the teaching and learning process (Wang et al., 2017; Chen et al., 2021). In this sense, subjects such as Chemistry and Biology, where abstract concepts are addressed, obtain extremely low grades from students, one of the main reasons being the lack of attention produced by little or no assimilation of their contents (Chen and Liao, 2015; Chen and Chen, 2018; Tarng et al., 2021).

On the other hand, the target groups resulting from the systematic review have been the 10 and 11th grade courses, which form the ages of 15–17 years (Paredes-Velastegui et al., 2018; Cen et al., 2020; Moreno-Guerrero et al., 2020; Koç et al., 2021; Lin et al., 2021). This shows that researchers seek to work with students who have the highest ages within the stage, the main reason being the high degree of disinterest that is reflected in that age group (Amores-Valencia and De-Casas-Moreno, 2019; González-Valenzuela and Martín Ruiz, 2019).

RQ2: What technological devices have been used to generate and/or execute AR applications?

Knowing the technological devices used during the execution of educational activities with Augmented Reality is essential, since it directly affects the possibilities and inconveniences that this type of scientific work can present. In this sense, it is necessary to have an electronic device, be it a tablet, computer or mobile phone to carry out the implementation of the investigations. Therefore, the results of this systematic review reflect that almost half of the investigations have used smartphones as a technological device. The main reason is that the students were in possession of one, giving them the opportunity to develop the activities designed individually, without the need to share devices (Paredes-Velastegui et al., 2018; Hsieh and Chen, 2019; Cen et al., 2020; Moreno-Guerrero et al., 2020; Koç et al., 2021; Tarng et al., 2021). This great inconvenience has been duly notified together with the problems of time availability in the Computer Classrooms and the Internet connection (Lin et al., 2015; Wang et al., 2017; Chen and Chen, 2018).

Regarding the software or applications used for the implementation of Augmented Reality, it can be seen how the applications created through the Vuforia platform, where the developers have been able to adapt the applications to the maximum, have been the most used, occupying the half of the research papers. In this sense, the development of own applications has prevailed, where it has been particularized according to the students who were going to develop each investigation. In this way, several aspects have been considered, such as their age, language and cognitive and sensory capacity (Wei et al., 2015; Paredes-Velastegui et al., 2018; Hsieh and Chen, 2019; Cen et al., 2020). It is important to highlight that the rest of the investigations, even having used a commercial application, have prepared the educational activities based on their own students.

RQ3: How are educational activities implemented with AR in the classroom?

All the researchers have designed or adapted the content for the different scientific works. This event shows that no teacher where these educational activities with Augmented Reality have been incorporated has been part of the process or design of the applications or software used. However, teachers have been immersed in some activities design processes, corroborating the inclusion of skills and learning standards appropriate to the subject and the educational context. Therefore, the professors who work day by day with the students who have undergone this research have played the role of guide and content designer, taking into account parameters of creativity and innovation (Wei et al., 2015; Wang et al., 2017; Chen and Chen, 2018; Paredes-Velastegui et al., 2018; Chen et al., 2020; Moreno-Guerrero et al., 2020). Likewise, the students have not been involved in the design process of the activities, however, they have developed an active, participatory and collaborative attitude. In research conducted by Fernández-Robles (2017) and Gallego-Pérez (2018) in university environments, it has been confirmed that the involvement of students in the creation of content favors motivation, since they have obtained better values in attention, relevance, confidence and satisfaction present in the ARCS model of Keller (1987, 2010).

Regarding the form of application of Augmented Reality, it has been based on the five dimensions exposed by Yuen et al. (2011), where the relevance of the context of the students when implementing said technology is showed. In this matter, object modeling is considered the form of application par excellence since it has been involved in practically half of the investigations. However, Augmented Reality books, gamification or skills training cannot be ruled out. This last process is used more in very advanced and specialized educational stages, since it supplies the means to acquire skills without harming any material or human damage (Rio-Guerra et al., 2019; Gómez-Tone et al., 2020).

RQ4: What motivational impact do students have based on the use of AR?

The research analyzed showed a comparative study between experimental groups and control groups. In such a way, that the control groups developed methodologies where Augmented Reality is not implemented as an educational resource, in contrast to the experimental groups. Based on the results obtained, it can be affirmed that the students of these experimental groups have ostensibly increased their motivation or interest during the educational activities with Augmented Reality. This assertion is determined by the results obtained, where specifically 83% of the studies analyzed have determined that the levels of motivation evaluated through questionnaires based on the parameters of attention, relevance, confidence and satisfaction established in the ARCS model of Keller (1987, 2010) have grown abruptly (Chen and Liao, 2015; Lin et al., 2015, 2021; Wei et al., 2015; Chen and Chen, 2018; Paredes-Velastegui et al., 2018; Cen et al., 2020; Chen et al., 2020; Moreno-Guerrero et al., 2020; Tarng et al., 2021).

On the other hand, the data obtained in the interviews or satisfaction questionnaires conducted a posteriori in the different investigations have endorsed the previous information, since most students have confirmed that the use of educational activities with Augmented Reality has fostered their predisposition learning, capturing their attention throughout the project. Therefore, this systematic review shows that the use of Augmented Reality in the classroom generates higher levels of motivation in students, and therefore they are of immense help for teaching. These results are like those achieved by Di Serio et al. (2013), Cabero-Almenara et al. (2017), and Gallego-Pérez (2018) in other educational stages.

RQ5: How does the use of AR influence the academic performance of students?

Based on the results obtained, it can be said that students show better grades when educational activities with Augmented Reality are implemented in the classroom. This assertion is preceded by the data extracted in the investigations, where a comparison between control groups and experimental groups has been conducted. For this, different tests or tests have been conducted, before and after the implementation of this educational technology, with the purpose of contrasting the results and refuting whether the academic performance in terms of qualifications has been increased thanks to the use of Augmented Reality. During the teaching-learning process. In this sense, it has been confirmed that two out of three students have seen their grades increase very significantly (Chen and Liao, 2015; Wei et al., 2015; Wang et al., 2017; Chen and Chen, 2018; Paredes-Velastegui et al., 2018; Cen et al., 2020; Moreno-Guerrero et al., 2020; Tarng et al., 2021).

Likewise, no research has emphasized that the use of Augmented Reality in the classroom has led to a decrease in the academic grades of students, so it is a sure bet of success (Lin et al., 2015, 2021; Hsieh and Chen, 2019; Chen et al., 2020; Koç et al., 2021). These results are like those obtained by Quintero et al. (2019) where it is showed that the use of Augmented Reality improved the performance of students with visual, motor, cognitive and auditory difficulties. It is necessary to point out that other research related to the field of medicine has confirmed that the use of Augmented Reality has ostensibly improved performance in terms of navigation screen control (Cagiltay et al., 2019; Jacobsen et al., 2019).

## Conclusion

This systematic review tries to value the relevance of ICT, specifically the use of Augmented Reality in the Secondary Education stage. According to (Unesco (2004), p. 30) “ICTs are a decisive tool to help students access vast resources of knowledge, collaborate with other classmates, consult experts, share knowledge, and solve complex problems using tools cognitive.” The use of ICT tools such as Augmented Reality that integrate open educational resources (OER) in an organic and transversal way in face-to-face, online and blended educational contexts, is a challenge for education, including Secondary Education (Unesco, 2019).

This educational stage, so problematic due to the low or null motivation that students present, together with poor academic results, is a great challenge for teachers (Picó-Lozano, 2014; Amores-Valencia and De-Casas-Moreno, 2019; González-Valenzuela and Martín Ruiz, 2019; Jerez-Carrillo, 2021). The first of the important aspects to know are the subject's where Augmented Reality has been introduced as an educational technology. In this sense, it has been found that the studies carried out by Chen and Liao (2015), Lin et al. (2015), Wang et al. (2017), Chen and Chen (2018), Hsieh and Chen (2019), Cen et al. (2020), Tarng et al. (2021), and have been developed in the areas of natural sciences and logical-mathematical. This shows that the use of Augmented Reality is more widespread in this field, as opposed to the areas of languages, arts or social sciences. Although the results analyzed in said investigations have confirmed that there is no significant difference regardless of the educational subject where it is implanted, the use of this educational technology must be addressed in the most and least attractive fields for the students, in this way could do a comparative study, and thus know the true potential and scope (Abad-Segura et al., 2020).

Regarding the target groups where this educational technology has been used, it has been an important turning point, since the ages where the greatest signs of disinterest and motivation appear are those between 12 and 18 years old (Amores-Valencia and De-Casas-Moreno, 2019; González-Valenzuela and Martín Ruiz, 2019). For this reason, this study has been conducted entirely dedicated to Secondary Education, since older students would give results conditioned by a more mature, more concentrated and motivated behavior, which would mean better academic performance (Cabero-Almenara et al., 2019a). Similarly, Primary Education students have elevated levels of attention and motivation, so their academic results tend to have high values (Toledo-Morales and Sánchez-García, 2017; Wang, 2017; Kirikkaya and Başgül, 2019; Lai et al., 2019; López-Belmonte et al., 2019). Within the Secondary Education stage itself, almost all the articles analyzed have been developed between the ages of 15 and 17, this range being the most conflictive in terms of lack of interest, motivation and low grades (Picó-Lozano, 2014; Jerez-Carrillo, 2021). For this reason, the investigations conducted by Cen et al. (2020), Koç et al. (2021), Lin et al. (2021), Moreno-Guerrero et al. (2020), and Paredes-Velastegui et al. (2018) have opted to analyze this age range.

In reference to the use of technological devices to bring the implementation of Augmented Reality to the classroom, the smartphone has been confirmed as the tool par excellence. This fact is due to the great limitations that educational centers present due to the unavailability of computers or tablets for each student, without forgetting the complicated situation of combining the use of these devices with other activities conducted in these centers (Vedadi et al., 2017). Thus, the availability of Computer Classrooms is extremely limited for many teachers due to the great demand received. In addition, it is acceptable to highlight the Internet connection problems present in educational centers. This translates into great concern for many teachers, since it shows that it is not exactly easy to integrate this educational technology in schools (Akçayir and Akçayir, 2017).

Once the technological devices have been addressed, software applications designed for the implementation of Augmented Reality should be looked for. In this aspect, two clearly found versions have been seen. The first refers to the applications created through the Vuforia platform, which have been duly studied and designed to fit perfectly in the students, taking into account aspects such as age, language, cognitive and sensory capacity of these (Wei et al., 2015; Paredes-Velastegui et al., 2018; Hsieh and Chen, 2019; Cen et al., 2020). About the second version, the materials created must largely adhere to the possibilities and limitations inherent in applications or software previously developed by other users. Based on this aspect, it can be affirmed that the contents developed and created by the researcher himself in full are more predisposed to success than the others (López-García et al., 2019).

The purpose of this research was to know the impact of the use of Augmented Reality in the Secondary Education stage based on motivation factors and academic performance. As for the data obtained from the different investigations addressed, they show an abrupt growth in the motivation levels of the students compared to the students who have not used this educational technology. This information has been reflected in the parameters of attention, relevance, trust and satisfaction analyzed following Keller's ARCS model (1987, 2010), which is a profound reason for the formation of teaching and learning practices that are based on the use of Augmented Reality (Chen and Liao, 2015; Lin et al., 2015, 2021; Wei et al., 2015; Wang et al., 2017; Chen and Chen, 2018; Paredes-Velastegui et al., 2018; Cen et al., 2020; Chen et al., 2020; Moreno-Guerrero et al., 2020; Tarng et al., 2021). In addition, the interviews conducted in several research works show a relationship between the use of this technology in the educational context and the increase in student motivation. This fact leads to highlight the importance of relying on the use of technologies, such as Augmented Reality, which enhance the interest of students (Keller, 2012).

Regarding the influence of this educational technology on the academic performance of the students reflected in their grades, the comparative results of the questionnaires or tests conducted show a significant difference between the students who made use of Augmented Reality in the teaching process and learning and those who tackled the activities without this technology. In this sense, the scores of the experimental groups were higher, which translates into an important reason to introduce Augmented Reality at these ages (Chen and Liao, 2015; Lin et al., 2015; Wei et al., 2015; Wang et al., 2017; Chen and Chen, 2018; Paredes-Velastegui et al., 2018; Cen et al., 2020; Chen et al., 2020; Moreno-Guerrero et al., 2020; Tarng et al., 2021). Likewise, a close relationship between motivation and academic performance is seen, since students with important levels of motivation obtain better grades (Hsieh and Chen, 2019; Koç et al., 2021; Lin et al., 2021). This information is valuable, since it is a great proposal for all teachers who want to see how the academic results of their students improve (Deigmann et al., 2015).

In relation to the limitations that have been seen, it should be noted that most of the research on Augmented Reality is not conducted in the Secondary Education stage, which has made it difficult to choose more research works. Likewise, most of these works do not jointly address the motivation and academic performance of students, and in turn, are studied from different perspectives. On the other hand, the works analyzed have not used a high number of students, due to the need to use technological devices. Regarding the possible lines of future research, it would be interesting to propose research where the teachers themselves are the designers and developers of software applications. However, this process is extremely complicated, since most teachers lack the knowledge and skills necessary to develop and apply this type of educational technology in the classroom (López-Belmonte et al., 2019). However, it can be said that students who have been immersed in the creation of Augmented Reality learning objects have shown much more satisfactory results, in terms of motivation and qualifications, than those who have only been consumers of this educational technology (Fernández-Robles, 2017; Quintero et al., 2019).

The present work aims to expand the current state of research in the field of Augmented Reality in the Secondary Education stage, grouping not only curricular aspects such as subjects, activities, method, but also two other major factors such as motivation and academic performance, with the purpose of capturing the repercussion of this educational technology for future studies.

## Data availability statement

The original contributions presented in the study are included in the article/Supplementary material, further inquiries can be directed to the corresponding author.

## Author contributions

AA-V, DB, and JB-B: conceptualization, design of the study, and writing—review and editing. AA-V: formal analysis, methodology, and writing—original draft. All authors approved the definitive version of the manuscript to be published.

## Conflict of interest

The authors declare that the research was conducted in the absence of any commercial or financial relationships that could be construed as a potential conflict of interest.

## Publisher's note

All claims expressed in this article are solely those of the authors and do not necessarily represent those of their affiliated organizations, or those of the publisher, the editors and the reviewers. Any product that may be evaluated in this article, or claim that may be made by its manufacturer, is not guaranteed or endorsed by the publisher.
